# Influence of Heterogeneous Karst Microhabitats on the Root Foraging Ability of Chinese Windmill Palm (*Trachycarpus fortunei*) Seedlings

**DOI:** 10.3390/ijerph17020434

**Published:** 2020-01-09

**Authors:** Yingying Liu, Xiaoli Wei, Zijing Zhou, Changchang Shao, Shicheng Su

**Affiliations:** College of Forestry, Guizhou University, Guiyang 550025, China; liuyingying2019@126.com (Y.L.); zhouzijing2019@163.com (Z.Z.); lying_127@163.com (C.S.); sushicheng2019@126.com (S.S.)

**Keywords:** karst microhabitats, Chinese windmill palm, root foraging, nutrient concentrations

## Abstract

Chinese windmill palms (*Trachycarpus fortunei*) are widely planted in karst bedrock outcrop areas in southwest China because of their high economic and ecological values. The aims of this study were to investigate the foraging ability of Chinese windmill palm seedlings planted in six different types of karst microhabitat and to identify the main environmental factors that influence root foraging ability. We planted three-year-old Chinese windmill palm seedlings in six typical karst microhabitats (i.e., rocky trough, rocky surface, rocky gully, rocky soil surface, rocky pit, and soil surface microhabitats). One year after transplanting, the seedlings were excavated to determine the morphological parameters values of new roots and the nutrient concentrations of new roots and leaves. The root foraging ability of Chinese windmill palm seedlings, defined as new root length and new root surface area, was significantly greater in the rocky trough, rocky soil surface, and soil surface microhabitats than in the rocky gully, rocky surface, and rocky pit microhabitats (*p* < 0.05). Redundancy analysis revealed that the main positive factor affecting the rooting ability of Chinese windmill palm seedlings was soil thickness. Chinese windmill palm seedlings improved their root absorption efficiency by increasing their root length and root surface area under soil nutrient deficiency conditions. The organic carbon, total nitrogen, and available potassium in soil positively influenced the concentration of N and K in roots. Total potassium in soil negatively influenced the biomass of new annual leaves and concentrations of N, P and K in new annual roots and leaves. Chinese windmill palm seedlings can be grown in the different karst microhabitats, especially in the rocky trough, rocky soil surface, and soil surface microhabitats, and, therefore, it is suitable for use in the regeneration of karst forests.

## 1. Introduction

Karst landforms account for 12%–15% of the global land system [1]. They are characterized as a large bedrock outcrop area with shallow soil, a discontinuous distribution, and complex and diverse micro-landforms. Previous studies have demonstrated a high diversity of microhabitats in karst regions [2], with diverse microclimatic characteristics in different microhabitats [3], and high spatial heterogeneity of soil nutrients, owing to uneven nutrient distribution and mobility [4,5], and of moisture [6,7].

Forest tree species that typically occur in karst environments show a variety of adaptive characteristics [8,9]. For example, some studies have reported that plants growing in shallow soils in karst regions can extend their roots vertically into the bedrock layers [10,11]. However, other studies have reported that in shallow soils in karst habitats, the vertical root growth of many plant species is highly constrained, and that plant roots typically extend horizontally rather than vertically [12,13]. The karst region is a peculiar rocky region. Roots of *Radermachera sinica*, which grew in loose rocky soils and exposed rocks, tapered rapidly, and the root extension was greater restricted, but roots of *Radermachera sinica* growing in shallow rocky soils tapered gradually, root extension was lightly restricted [13]. In karst region, root growth is mainly constrained by the decline in soil thickness [14]. The foraging behavior of plant roots is not only influenced by the soil conditions but also by the genetic endogenous programs of the plant. Generally, plants increase their root length and root tips to seek nutrients in nutrient-limited rocky soils [15]. Under heterogeneous soil conditions, plants activate foraging responses to enhance their nutrient uptake efficiency. These include morphological changes, such as root proliferation when encountering nutrient-rich soil patches [16], modifying their root system architecture [17], and increasing their root biomass [18]; physiological changes that enhance their nutrient uptake ability; and rhizosphere microorganism changes, such as the formation of symbiotic arbuscular mycorrhizal associations [19,20,21]. These foraging strategies may be an adaptation to the spatial heterogeneity of nutrient availability. Root traits affect nutrient uptake and, consequently, the nutritional status of the plant [22]. The accumulation and distribution of N, P, K and other elements in plants are determining factors for plant growth and, therefore, studying the accumulation and distribution of nutrients is important for forest management. One study suggested that, in a non-competitive environment, nutrient heterogeneity and fertilization had no effect on the fine-root morphology of spruce seedlings but did increase the accumulation of P and K in fine roots [23]. Currently, we have a poor understanding of the influence of root foraging and nutrient accumulation on the growth and survival of seedlings transplanted in highly heterogeneous karst microhabitats, despite their important roles in the restoration and reconstruction of vegetation in karst areas.

To investigate the root foraging ability of transplanted seedlings in karst areas, we used Chinese windmill palm (*Trachycarpus fortunei*) seedlings because Chinese windmill palm is a pioneer plant and is the main tree species used for vegetation restoration in karst habitats in the ecologically fragile rocky mountain areas of southwest China. These Chinese windmill palms are widely planted owing to their high economic value and their ecological role in protecting the soil from erosion and their efficient conservation of water. Under natural conditions, Chinese windmill palms are distributed over different karst habitats and show extremely strong vitality. The seeds of mature Chinese windmill palm trees disperse into different microhabitats after maturation and can survive tenaciously in various microhabitats. Chinese windmill palms are shallow-rooted plant species, with well-developed fibrous roots, and without main root [24]. We hypothesized that the root system of Chinese windmill palms spreads widely in the topsoil to absorb more nutrients, so root foraging ability is mainly constrained by the soil nutrients, instead of soil thickness, and the ability of new root proliferation is enhanced at nutrient-limited soils. In this study, Chinese windmill palm seedlings were planted in six different karst microhabitats to assess the foraging ability of seedlings and to determine which environmental factors are the main factors influencing foraging, so as to provide insight and practical advice for the silviculture of Chinese windmill palm seedlings in karst regions. In our study, the root foraging ability of Chinese windmill palm seedlings was expressed by the new root biomass and new root morphology parameters (including root length and root surface area) [17].

## 2. Materials and Methods

### 2.1. Study Region

**Figure 1:**
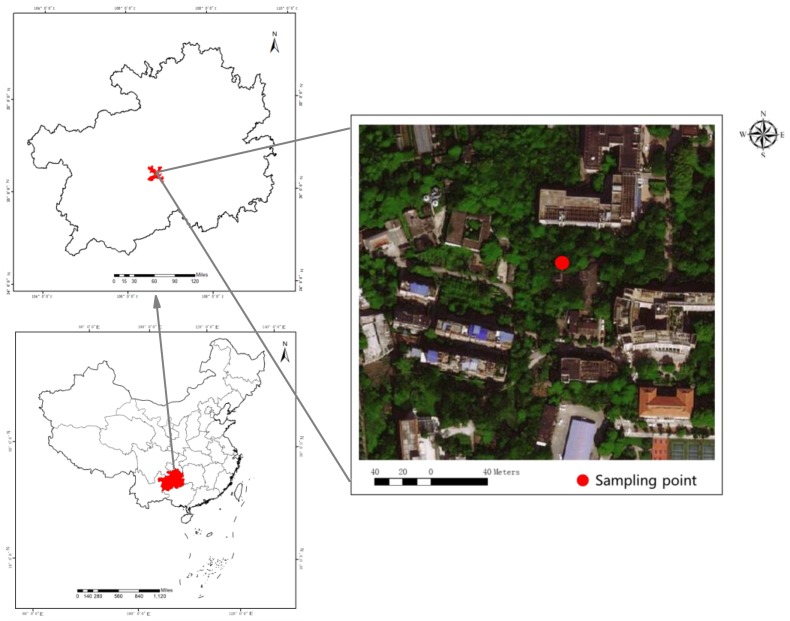
Location of the study area in the South Campus of Guizhou University in Huaxi District, Guiyang City, Guizhou Province, China.

The experimental field was located in the South Campus of Guizhou University in Huaxi District, Guiyang City, Guizhou Province (26°25′25″–26°25′26″ N, 106°40′12″–106°40′13″ E) (Figure 1). This region is characterized by a mid-subtropical plateau monsoon climate, with heavy rain in spring and summer, an annual average temperature of 15.8 °C, an annual precipitation of 1229 mm, an annual average relative humidity of 79%, a total solar radiation of 3567 MJ/m^2^, and an annual growth period of 271 days. According to the criteria used by Zhu [25] to classify microhabitat types, we identified six types of karst microhabitat at the experimental field site: (I) a rocky trough (i.e., the bedrock protrudes horizontally to form a semi-closed strip-like fissure with a soil area of 0.1–1 m^2^); (II) a rocky surface (i.e., humus gather on the surface); (III) a rocky gully (i.e., a continuous corrosion gully or erosion gully of the outcropped bedrock with a depth:width ratio of usually less than 2, wide openings and U-shaped cross-sections); (IV) a rocky soil surface (i.e., a small tableland with more than 30% of the bedrock exposed, similar to the soil surface); (V) a rocky pit (i.e., an enclosed corrosion depression of the outcropped bedrock and a soil area of 0.1–1 m^2^); and (VI) soil surface (i.e., a continuous soil surface with a length and width greater than 2 m).

### 2.2. Plant Materials

Three-year-old Chinese windmill palm seedlings from Huaxi district in Guizhou Province were used as the plant material in this study.

### 2.3. Sampling Design

**Figure 2:**
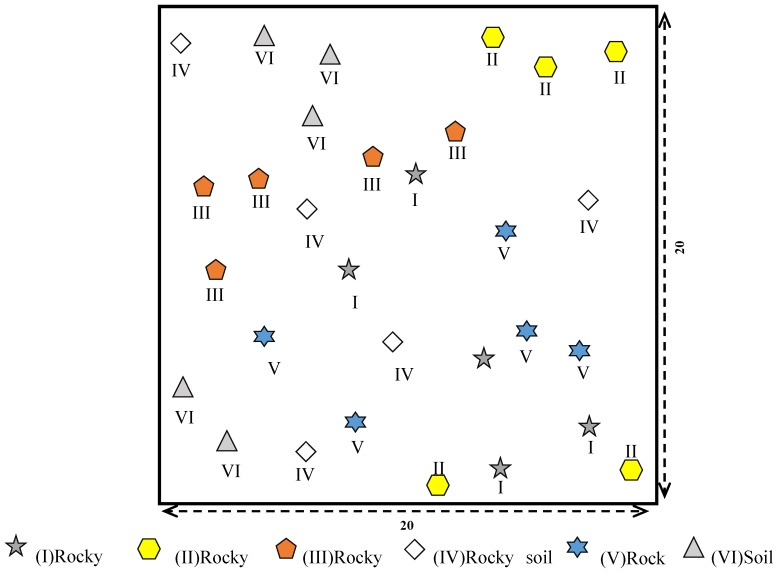
Sampling design, with six microhabitats: (I) rocky trough, with a soil area of 0.1–1 m^2^; (II) rocky surface, with a soil area of 0.04–0.3 m^2^; (III) rocky gully, with a soil area of 0.02–0.4 m^2^; (IV) rocky soil surface, with a soil area of 1–3 m^2^; (V) rocky pit, with a soil area of 0.1–1 m^2^; and (VI) soil surface, with a soil area of 4–6 m^2^. *n* = 5.

A 20 m × 20 m sample plot with six microhabitat types (I–VI; see description in the ‘Study region’ section above) was set up on a karst landform developed by dolomite corrosion and erosion in the South Campus, with five replicates of each microhabitat type (Figure 2).

In December 2016 (i.e., in the winter of 2016), three-year-old Chinese windmill palm seedlings, with an average initial plant height, ground-line diameter and biomass of 52.79 ± 5.84 cm, 11.75 ± 1.25 mm and 19.58 ± 4.25 g respectively, were selected. Rotten or excessively long roots were pruned to ensure that each seedling used in the experiment had 13.00 ± 3.00 healthy roots, with primary roots that were 10.00 ± 2 cm in length. In the winter of 2016, about 4–6 seedlings were planted in each of five replicate microhabitats following the dibble planting method (i.e., 25 seedlings were planted in each of the six microhabitat types, i.e., 150 seedlings in total) and allowed to grow naturally. In December 2017, three seedlings in each of five replicate microhabitats (i.e., fifteen independent seedlings were excavated in each type microhabitat) were randomly selected, and whole seedlings (i.e., roots, stem, and leaves) were excavated for further analysis.

### 2.4. Measurement of Root Morphological Indicators

One seedling of three excavated seedlings in each of five replicate microhabitats (i.e., five independent seedlings were excavated in each type microhabitat) was randomly selected for root parameter analysis. Plant roots were washed using the commercially available Root Washing System (Bio-Equip, #QT-RWC, Channel Technology Group Limited, Beijing, China). After the soil was removed, the roots were rinsed with distilled water and the surface water was absorbed with clean absorbent paper. New roots that had developed during 2017 were distinguished from old roots according to color and surface texture [26]. New roots were cut and scanned using a root scanner (ScanMaker i800 Plus, Wseen Detection Technology Co., Ltd., Hangzhou, China). Scanned images were saved and a quantitative analysis of the root morphological parameters was performed using a Wseen LA-S Root Analysis System (Wseen Detection Technology Co., Ltd., Hangzhou, China). New roots were oven-dried at 105 °C for 30 min to deactivate enzymes and then dried at 60 °C until a constant mass was obtained, and their weight was recorded.

### 2.5. Measurement of Root and Leaf Biomass and Nutritional Components

All roots and leaves of two other seedlings, excavated from each of five replicate microhabitats, were rinsed with tap water and then with distilled water. Surface water was absorbed with clean absorbent paper. New annual roots and leaves were cut and weighed, oven-dried at 105 °C for 30 min to deactivate enzymes, and then dried at 60 °C to constant mass and their weight was recorded. Three seedlings, including one for root analysis and the other two (i.e., fifteen independent seedlings were excavated from each type microhabitat) were used to analysis biomass, nutrient of new roots and leaves.

All three seedlings from each microhabitat were combined for nutrient analyses. Root and leaf samples of the aforementioned three seedlings were sieved through a 2-mm mesh strainer and digested with H_2_SO_4_-HClO_4_ on a thermostat heating plate to measure the concentrations of nitrogen, potassium, and phosphorus. Total nitrogen was determined according to the indophenol blue method [27], and the absorbance value was detected at 625 nm using a UV-2600 spectrophotometer (Shimadzu Corp., Tokyo, Japan); total potassium was determined by FP6410 flame photometry (Precision Scientific Instrument Co., Ltd., Shanghai, China) [28]. Total phosphorus was determined using the Mo-Sb colorimetric method [29], the absorbance value was assayed at 700 nm using a UV-2600 spectrophotometer (Shimadzu Corp., Tokyo, Japan).

### 2.6. Determination of Soil Physical and Chemical Characteristics

Removing the litter from soil surface, one undisturbed soil samples were extracted from each of five replicate microhabitats (i.e., five independent undisturbed soil samples were collected in each type microhabitat), using a soil core (100 cm^3^), to estimate water content and soil bulk density (g cm^−3^) based on total soil weight (g) and the volume of the cylinder [30]. Then, one soil profile was sampled in each of five replicate microhabitats (i.e., five independent undisturbed soil samples were sampled in each type microhabitat). Every combined soil sample was collected from each soil profile, and was stored in plastic bags, taken to the laboratory, air-dried at room temperature, and sieved through a 2-mm mesh. Sieved samples were used to determine soil organic carbon, total nitrogen, total phosphorous, total potassium, hydrolyzable nitrogen, available phosphorous, and available potassium. It should be noted that combined soil samples were collected from the top 0–40 cm soil layer in each of five replicate microhabitats (i.e., five independent samples were collected in each type microhabitat). If the soil thickness at the point of collection was less than 40 cm, sampling was performed according to the actual soil thickness. Sampling was only conducted after three consecutive sunny days following any rain to ensure an accurate reflection of the soil bulk density and porosity (rain can increase soil bulk density and reduce porosity).

Soil organic carbon was measured using the potassium dichromate volumetric method (Tiurin’s method) [31], which involved heating samples with a dichromate-sulfuric acid solution using an oil bath, and then titration with a standard solution of ammonium ferrous sulfate. Total nitrogen was determined using the semi-micro Kjeldahl method [32], which involved digestion with H_2_SO_4_–HClO_4_ on a heating plate and diffusion by alkali hydrolysis in a constant-temperature incubator. Hydrolyzable nitrogen was measured using the semi-micro Kjeldahl method [32], with diffusion by alkali hydrolysis in a constant temperature incubator. Total phosphorus was determined using the Mo-Sb colorimetric method [29], which involved digestion with H_2_SO_4_–HClO_4_ on a heating plate, absorbance development, and measurement at 700 nm using a UV-2600 spectrophotometer (Shimadzu Corp., Tokyo, Japan). Available phosphorus was determined using the Mo-Sb colorimetric method [29], which involved extraction with sodium bicarbonate on an oscillator and detection of the absorbance value at 700 nm using a UV-2600 spectrophotometer. Total potassium was measured using an FP6410 flame photometer (Precision Scientific Instrument Co., Ltd., Shanghai, China) after digesting samples with H_2_SO_4_–HClO_4_ on a heating plate. Available potassium was determined using an FP6410 flame photometer [28] after extraction by ammonium acetate ions. Specific gravity was determined using the pycnometer method [33].

Porosity was calculated according to the bulk density and specific gravity of the soil [34], and calculated as follows:

$$\mathrm{Porosity}=1-\frac{\mathrm{Bulk}\;\mathrm{Density}}{\mathrm{Specific}\;\mathrm{Gravity}}\times 100\%.$$

### 2.7. Data Analysis

Statistical analysis of the data was performed using SPSS25.0 (SPSS Inc., IBM Corporation, Chicago, IL, USA). Before performing an analysis of variance (ANOVA), the root morphological data, biomass, nutrient accumulation data and the physical and chemical data of soil from different karst microhabitats were assessed for homogeneity of variance by performing a Levene’s test. The biomass data showed homogeneity of variance; however, all other data showed unequal variance. So, non-parametric tests were used in this study. A Kruskal–Wallis test was used to analyze the data of soil characteristics, root morphological characteristics, biomass and nutrient concentrations of new roots and leaves, followed by stepwise step-down for multiple comparisons. *p* ≤ 0.05 was considered to be statistically significant. The physical and chemical soil characteristics were expressed as the median ± the range. Box plots of root morphological characteristics, biomass and nutrient concentrations were generated using Origin2018 (OriginLab, Northampton, MA, USA).

Redundancy analysis (RDA) was used to assess the variation of response variables, using data multivariate analysis in Canoco for Windows 4.5 (Microcomputer Power, Ithaca, NY, USA). Two RDAs were run to assess the association between (1) environmental variables and root morphological characteristics, and (2) environmental nutrient variables and biomass and nutrient accumulation in roots and leaves. Variables were selected manually under an unrestricted model with Monte Carlo permutation tests (9999 permutations). Environmental factors were run using forward selection to check whether they were statistically significant, and environmental factors of statistical importance were used in redundancy analyses.

## 3. Results and Analysis

### 3.1. Soil Characteristics

The soils in the six karst microhabitats (i.e., rocky trough, rocky surface, rocky gully, rocky soil surface, rocky pit, and soil surface) investigated in this study were highly heterogeneous (Table 1). Rocky trough (I), rocky soil surface (IV), and soil surface (VI) microhabitats were characterized by thicker soil, moderate water content, and moderate porosity. The nutrient concentrations in soil in rocky trough (I), rocky gully (III) and rocky soil surface (IV) microhabitats was low. In the rocky surface (II) microhabitat, the soil layer was shallow with a humus soil, high nutrient concentrations, and high porosity. This soil loses water quickly and becomes dry on sunny days. The rocky gully (III) microhabitat is a continuous corrosion gully or erosion gully of the outcropped bedrock, with a water source that is mainly from lateral seepage movement and is wet all the year round. The soil moisture content was high, with a large volumetric weight and small porosity. The rocky pit (V) microhabitat was an enclosed corrosion depression of the outcropped bedrock and, therefore, loses water on sunny days.

### 3.2. Morphological Characteristics of New Roots

**Figure 3:**
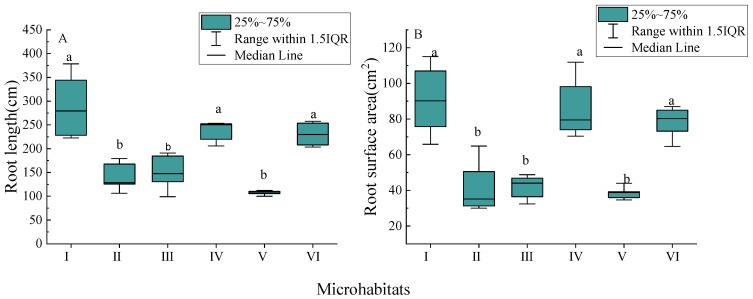
Morphological characteristics of new annual roots of Chinese windmill palm seedlings planted in different microhabitats: (**A**) root length; (**B**) root surface area. Data are presented as box plots: boxes represent the 25th to 75th percentiles, lines within the boxes represent the median, and lines outside the boxes represent values within 1.5 times interquartile range (IQR). Different lower-case letters (a, b) indicate significant differences at *p* < 0.05.

Parameters values for root length and root surface area of Chinese windmill palm seedlings planted in rocky trough (I), rocky soil surface (IV), and soil surface (VI) microhabitats were significantly higher than those for seedlings planted in rocky surface (II), rocky gully (III), and rocky pit (V) microhabitats (*p* < 0.05; Figure 3 and Figure S1). This was especially notable for the rocky trough (I) microhabitat, which had the highest root morphological parameters (median ± range): root length, 279.32 ± 155.74 cm; root surface area, 90.13 ± 49.18 cm^2^.

### 3.3. Biomass of New Annual Roots and Leaves

**Figure 4:**
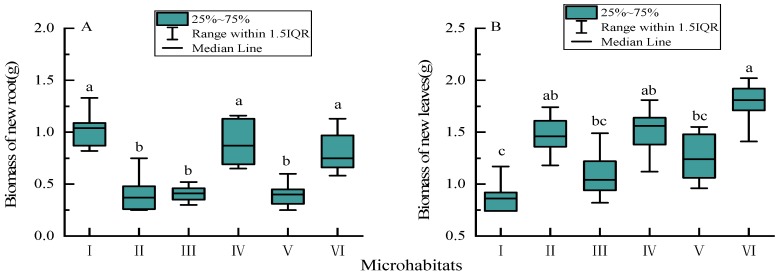
Biomass of new annual roots (**A**) and leaves (**B**) of Chinese windmill palm seedlings planted in different microhabitats. Data are presented as box plots: boxes represent the 25th to 75th percentiles, lines within the boxes represent the median, and lines outside the boxes represent values within 1.5 times interquartile range (IQR), little black-boxes within the boxes represent the means. Different lower-case letters (a–c) indicate significant differences at *p* < 0.05.

The root biomass of Chinese windmill palm seedlings planted in rocky trough (I), rocky soil surface (IV), and soil surface (VI) microhabitats was significantly greater than that of seedlings planted in rocky surface (II), rocky gully (III), and rocky pit (V) microhabitats (*p* < 0.05; Figure 4). This was particularly notable for seedlings planted in the rocky trough (I) microhabitat, which had the largest root biomass (median ± range: 1.04 ± 0.51 g). By contrast, the leaf biomass of seedlings planted in microhabitat I was the lowest (median ± range: 0.86 ± 0.43 g) and significantly different to the leaf biomass of seedlings planted in rocky surface (II), rocky soil surface (IV), and soil surface (VI) microhabitats (*p* < 0.05). Seedlings planted in the soil surface (VI) microhabitat produced significantly greater leaf biomass (median ± range: 1.81 ± 0.61 g) than those planted in rocky trough (I), rocky gully (III) or rocky pit (V) microhabitats (*p* < 0.05).

### 3.4. Nutrient Concentrations of New Annual Roots and Leaves

**Figure 5:**
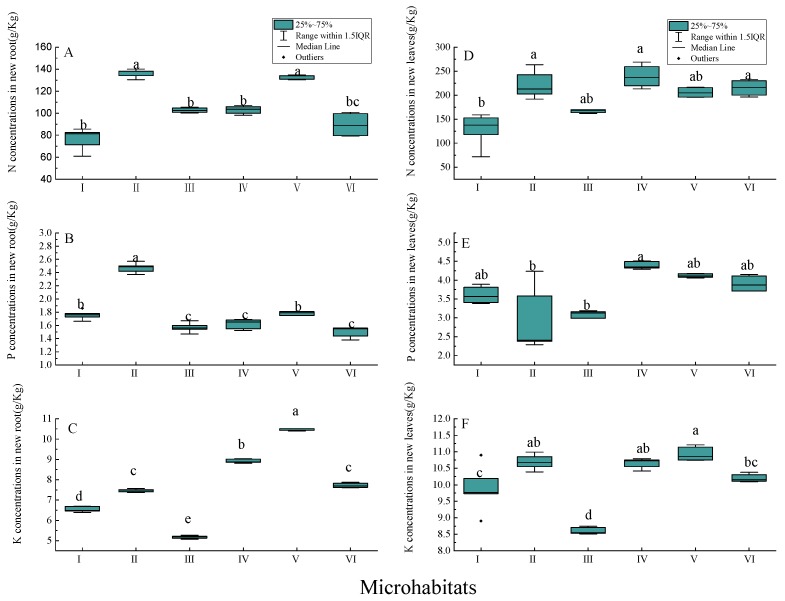
Nutrient concentrations (g/kg) in new annual roots and leaves of Chinese windmill palm seedlings planted in different microhabitats: (**A**) N in roots; (**B**) P in roots; (**C**) K in roots; (**D**) N in leaves; (**E**) P in leaves; and (**F**) K in leaves. Data are presented as box plots: boxes represent the 25th to 75th percentiles, lines within the boxes represent the median, and lines outside the boxes represent values within 1.5 times interquartile range (IQR). Different lower-case letters (a–e) indicate significant differences at *p* < 0.05.

There was a significant difference in the accumulation of N, P, and K in new roots of Chinese windmill palm seedlings when grown in different karst microhabitats (Figure 5). N and P concentrations were highest in the roots of seedlings planted in microhabitat II: 138.01 ± 9.69 g/kg (median ± range) and 2.49 ± 0.2 g/kg (median ± range), respectively. With the exception of N accumulation in the new roots of seedlings planted in microhabitat V, the accumulation of N and P in new roots of seedlings planted in the other microhabitats were not significantly different (*p* > 0.05). The K concentration in new roots of seedlings planted in microhabitat VI was significantly higher than that in seedlings planted in the other five microhabitats (*p* < 0.05).

### 3.5. Redundancy Analysis

**Figure 6:**
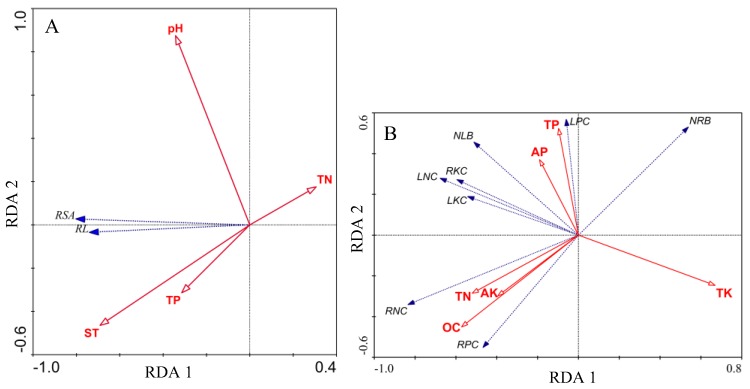
Biplots of redundancy analysis to show associations between environmental variables and root morphological characteristics (**A**) and between nutrient concentrations in soil and biomass and nutrient concentrations of new roots and leaves of Chinese windmill palm seedlings (**B**). Arrows pointing in the same direction indicate variables that are positively correlated whereas arrows pointing in opposite directions indicate variables that are negatively correlated. Abbreviations: AK, available potassium; AP, available phosphorus; LKC, leaf potassium concentrations; LNC, leaf nitrogen concentrations; LPC, leaf phosphorus; NLB, new leaves biomass; NRB, new roots biomass; OC, organic carbon; pH, potential of hydrogen; RKC, root potassium concentrations; RL, root length; RNC, root nitrogen concentrations; RPC, root phosphorus concentrations; RSA, root surface area; ST, soil thickness; TK, total potassium; TN, total nitrogen; TP, total phosphorus.

A biplot of the redundancy analysis associating root morphological characteristics with environmental factors of statistical importance is shown in Figure 6A. The most significant variables obtained using forward stepwise selection were total nitrogen, total phosphorus, potential of hydrogen, and soil thickness (Table 2). The species–environmental correlations for the first and second axis were 0.788 and 0.169, respectively (Table 2). The first and second axes explain 59.9% and 0.1% of the variance, respectively (Table 2). The first RDA axis correlated with a combination of total nitrogen, total phosphorus, pH and soil thickness (Figure 6A, Table 2). The first axis explained the majority of the variation in root morphology. Variance explained by all soil variables was 81%. Soil thickness accounted for the largest statistically significant amount of variation and explained 28.70% of the variance in root morphological characteristics (Monte Carlo permutation test with 9999 permutations, *p* = 0.0014).

A biplot of the redundancy analysis associating biomass and the nutrient concentrations of new roots and leaves with statistically important environmental factors is shown in Figure 6B. The most significant variables obtained using forward stepwise selection were organic carbon, total nitrogen, total phosphorus, available phosphorus, total potassium, and available potassium (Table 3). Species–environmental correlations for the first and second axis were 0.906 and 0.808, respectively (Table 3). The first and second axes explained 39.3% and 15.5% of the variance, respectively (Table 3). The first RDA axis correlated with a combination of organic carbon, total nitrogen, total potassium, and available potassium. The second RDA axis correlated with organic carbon, total nitrogen, total phosphorus, available phosphorus, total potassium, and available potassium (Figure 6B, Table 3). Together, soil nutrient variables explained 69% of the variance in biomass and the nutrient concentration of roots and leaves. Total potassium accounted for the largest statistically significant amount of variation. Total potassium explained 20.89% of the variance in biomass and nutrient concentration of roots and leaves (Monte Carlo permutation test with 9999 permutations, *p* = 0.0001).

## 4. Discussion

### 4.1. Root Morphological Characteristics of Palm Seedlings in Different Karst Microhabitats

Plants and their associated soil environments are interrelated and interact with each other in ecosystem development [35,36]. The soil layer is shallow in karst regions and horizontal variations in soil properties are more prominent than vertical variations [37]. The heterogeneity of soil is mainly affected by the spatial variation of microhabitats [37] and, therefore, the utilization of different microhabitats by plants is distinct [38]. Root morphology can influence the nutrient foraging strategies of plants [39]. In the present study, root regeneration of Chinese windmill palm seedlings was poor in rocky surface, which supports previous findings that root growth and root extension are restricted in exposed rocks [13], however, root regeneration of Chinese windmill palm seedlings was poor in rocky surface, which do not support previous observations that roots growth was lightly restricted in shallow rocky soils (<30 cm) [13].The root foraging intensity of Chinese windmill palm seedlings after transplantation was significantly stronger in rocky trough, rocky soil surface, and soil surface microhabitats than in rocky gully, rocky surface, and rocky pit microhabitats (*p* < 0.05). These findings are not consistent with findings reported by Matthes-Sears et al. [40] that root growth of *Thuja occidentallis*, with the same age (>6 years old), was no significant difference in different Karst microsite characteristics. Compared with the period when trees grow naturally in different karst habitats for many years, the factors in different microhabitats may be more important at earlier life stage of plants, especially at the early stage of transplantation. Studying the root growth of Chinese windmill palm seedlings after transplanting can effectively assess its adaptability to the divers microhabitats, and then provide insight and practical advice for the silviculture of Chinese windmill palm seedlings in karst regions.

### 4.2. Biomass and Nutrient Concentrations of New Annual Roots and Leaves in Different Karst Microhabitats

The growth of new roots and leaf of Chinese windmill palm seedlings after they were transplanted to karst microhabitats were the result of interactions between the Chinese windmill palm itself and environmental factors, reflecting not only the survival ability of the plant, but also the adaptability of the plant to environmental conditions. Martinkova et al. [41] reported that the accumulated root biomass was greater in plants germinating in nutrient-rich soil patches compared with those germinating in nutrient-deficient patches. However, in the present study, although rocky surface microhabitat was rich in soil nutrients, it had a shallow, loose soil and low water content, which may partly explain why the new root-generating capacity was weak after transplanting, with low root biomass accumulation.

In the present study, root nutrient concentration was mainly affected by soil nutrient concentration, which is consistent with conclusions reported by Li et al. [23]. The roots of seedlings that were planted in rocky surface and rocky pit, where soil samples had significantly higher levels of hydrolyzed nitrogen (*p* < 0.05), had higher N concentration than seedlings planted in other microhabitats. However, the N concentration of new leaves in rocky surface and rocky pit did not have significantly difference to that of leaves in rocky gully, rocky soil surface and soil surface. These findings do not support previous observations of a positive correlation between N concentration in leaves and N concentration in roots [42].

### 4.3. Relationship between Root Morphology of Palm Seedlings and Environmental Factors

The RDA ordination effectively identified the importance of environmental variables in influencing new root growth, and soil thickness, available potassium, available phosphorus and total nitrogen all affected root foraging ability of Chinese windmill palm seedlings, variance explained by statistically important environmental factors was 65.34%. Soil thickness was the main environmental factor influencing root growth, explaining 28.7% of the variance in root morphological characteristics. The thicker the soil layer, the better the root growth, suggesting that soil thickness might play a key role in Chinese windmill palm seedling regeneration. This result is consistent with findings reported by Lin et al. that soil thickness was the main influence of topography on seedling regeneration in a tropical karst forest, likely because of its effects on soil moisture availability [43]. However, this result is not consistent with findings reported by Matthes-Sears et al. [40] that root growth of *Thuja occidentallis*, with the same age (>6 years old), was no significant difference in presence or absence of soil. It is possible that the dependence of plants on different microhabitats declines as they increase in size for its exploiting environments beyond the immediate germination site, for example rooting in rock. Total nitrogen and available potassium of the soil in diverse microhabitats were negatively correlated with the root foraging ability of Chinese windmill palm seedlings. The root foraging ability of Chinese windmill palm seedlings was strong in low nutrient concentrations. This supports previous field observations that under conditions of soil nutrient deficiency, plants can improve their root absorption efficiency by increasing their root length [44]. However, nutrient deficiencies in the soil cannot be offset simply by increased root growth. In the present study, seedlings that were planted in the rocky trough microhabitat, which had a low soil nutrient concentration, had the lowest new leaf biomass, indicating that, when nutrients are limited, plants transfer more resources to the root system, which has a negative impact on the growth of its aboveground parts. This result is consistent with the previous study that when woody plants invest more resources in their root system in response to nutrient limitation, resource allocation to leaves is reduced [45]. Therefore, based on the results, the cultivation of an aggregating topsoil to thicken the soil layer is worth considering when planting Chinese windmill palm seedlings in karst forests. (The cultivation of an aggregating topsoil is a method of afforestation commonly used in karst areas with shallow soil layers. Soil in the vicinity of the planting hole is gathered up around the planting hole to increase the thickness of the soil layer).

The RDA ordination effectively identified the importance of environmental variables in influencing the biomass and nutrient concentrations of new annual roots and leaves of Chinese windmill palm seedlings planted in different microhabitats, and organic carbon, total nitrogen, total phosphorus, available phosphorus, total potassium, and available potassium all affected root foraging ability of Chinese windmill palm seedlings, variance explained by statistically important environmental factors was 67.6%. We find that total potassium in soil was a negative factor affecting the biomass of new annual leaves and nutrient concentrations of new annual roots and leaves. Total potassium in soil played more important role than nitrogen and phosphorus on growth and yield of Chinese windmill palm fiber [46]. Potassium was a positive factor affecting plant fiber yield [47]. Four-year-old Chinese windmill palm seedlings began to yield fibers from the lignified leaf sheath, the high concentration of total potassium in soil likely improved the fiber yield, so more nutrients were distributed to form fibers, eventually leading to a decrease in biomass and nutrient concentration of new root and new leaf.

## 5. Conclusions

The root-foraging ability of Chinese windmill palm seedlings, defined as new root length and new root surface area, was greater in rocky trough, rocky soil surface, and soil surface microhabitats than in rocky gully, rocky surface, and rocky pit microhabitats. Even in soils with low nutrient concentrations, the roots of Chinese windmill palm seedlings grew well. Rooting foraging ability of Chinese windmill palm seedlings was mainly positively affected by soil thickness. Under soil nutrient deficiency, Chinese windmill palm seedlings enhanced root absorption efficiency by increasing their root length and root surface area. In heterogeneous microhabitats, the organic carbon, total nitrogen, and available potassium in soil positively affected the concentration of N and K in roots, and total potassium in soil negatively affected the biomass of new annual leaves and concentrations of N, P and K in new annual roots and leaves.

## Figures and Tables

**Table 1:** Principal physical and chemical characteristics of soils in different microhabitats (n = 5).

Microhabitat	Rocky Trough (I)	Rocky Surface (II)	Rocky Gully (III)	Rocky Soil Surface (IV)	Rocky Pit (V)	Soil Surface (VI)
Soil thickness (cm)	54.33 ± 6.03 a	19.67 ± 2.08 c	41.00 ± 2.65 b	39.67 ± 3.06 b	38.00 ± 2.00 b	44.33 ± 4.51 ab
Volumetric weight (g/cm^3^)	1.56 ± 0.13 a	1.36 ± 0.43 b	1.63 ± 0.04 a	1.54 ± 0.09 a	1.44 ± 0.09 b	1.58 ± 0.11 a
Specific gravity (g/cm^3^)	2.53 ± 0.05 a	2.35 ± 0.02 b	2.51 ± 0.02 a	2.45 ± 0.04 a	2.51 ± 0.06 a	2.55 ± 0.02 a
Porosity (%)	37.19 ± 1.15 bcd	45.47 ± 8.21 a	34.92 ± 1.37 d	36.77 ± 0.97 cd	42.16 ± 1.30 ab	38.47 ± 1.72 bc
Water content (%)	39.11 ± 1.99 bc	37.62 ± 9.06 bcd	41.33 ± 2.19 a	36.51 ± 1.04 cd	32.08 ± 6.52 d	40.82 ± 1.76 ab
Potential of hydrogen	7.11 ± 0.08 abc	7.15 ± 0.11 ab	7.00 ± 0.06 bc	7.28 ± 0.16 a	6.96 ± 0.06 c	6.94 ± 0.06 c
Organic carbon (g/kg)	7.19 ± 0.34 c	13.86 ± 0.15 a	5.58 ± 0.55 e	6.91 ± 0.19 d	8.58 ± 1.14 b	6.43 ± 0.74 d
Total nitrogen (N%)	0.2 ± 0 b	0.34 ± 0.04 a	0.14 ± 0.02 c	0.18 ± 0.04 b	0.2 ± 0.02 b	0.18 ± 0.03 b
Hydrolyzed nitrogen (mg/kg)	112.74 ± 2.15 d	185.5 ± 73.99 a	87.41 ± 7.28 e	125.91 ± 6.25 c	131.99 ± 10.34 b	116.18 ± 15.87 d
Total phosphorus (g/kg)	0.13 ± 0.02 c	0.22 ± 0.03 b	0.11 ± 0.01 d	0.13 ± 0	0.11 ± 0.01 d	0.46 ± 0.05 a
Available phosphorus (mg/kg)	0.62 ± 0.14 d	2.5 ± 0.1 b	0.4 ± 0.12 e	0.21 ± 0.07 f	0.82 ± 0.16 c	5.24 ± 0.07 a
Total potassium (g/kg)	8.99 ± 0.91 a	8.17 ± 0.04 ab	7.96 ± 0.64 b	6.85 ± 0.12 c	6.45 ± 0.11 d	8.66 ± 2.21 ab
Available potassium (mg/kg)	103.47 ± 1.01 d	138.86 ± 1.16 b	89.94 ± 2.51 e	52.45 ± 0.63 f	160.44 ± 1.62 a	135.49 ± 2.87 c

Above values shown represent the median value ± the range. Different lower-case letters (a–f) within a row for each experimental factor indicate significant differences at *p* < 0.05.

**Table 2:** Results of redundancy analysis variance partitioning of environmental factors with morphological characteristics of new annual roots of Chinese windmill palm seedlings planted in different microhabitats.

Item	Axis 1	Axis 2	Axis 3	Axis 4	Total Variance
Eigenvalues	0.599	0.001	0.370	0.030	1
Species–environment correlations	0.788	0.169	0	0	
CV of species data (%)	59.9	60.0	97.0	100	
CV of species–environment relationship (%)	99.8	100	0	0	
Sum of all eigenvalues					1
Variance explained by all variables					0.817
Variance explained by selected variables					0.6534
Forward selection of variables	RDA 1	RDA 2	*F* value	*p* value	Extra fit
Soil thickness (cm)	−0.5449	−0.0787	11.27	0.0014 **	0.287
Potential of hydrogen	−0.2701	0.1480	7.41	0.0094 **	0.1535
Total phosphorus (g/kg)	−0.2478	−0.0532	9.98	0.0025 **	0.1552
Total nitrogen (N%)	0.2411	0.0297	4.64	0.0352 *	0.0577

CV, cumulative variance. * Significant at *p* < 0.05; ** significant at *p* < 0.01.

**Table 3:** Results of redundancy analysis variance partitioning of environmental factors with the biomass and nutrient concentrations of new annual roots and leaves of Chinese windmill palm seedlings planted in different microhabitats.

Item	Axis 1	Axis 2	Axis 3	Axis 4	Total Variance
Eigenvalues	0.393	0.155	0.077	0.042	1
Species–environment correlations	0.906	0.808	0.762	0.823	
CV of species data (%)	39.3	54.7	62.5	66.7	
CV of species–environment relation (%)	58.1	81	92.4	98.7	
Sum of all eigenvalues					1
Variance explained by all variables					0.69
Variance explained by selected variables					0.676
Forward selection of variables	RDA 1	RDA 2	*F* value	*p* value	Extra fit
Organic carbon (g/kg)	−0.5188	−0.3644	8.91	0.0001 **	0.1963
Total nitrogen (N%)	−0.4716	−0.2308	3.97	0.0042 **	0.0653
Total phosphorus (g/kg)	−0.0882	0.4232	6.44	0.0001 **	0.1181
Available phosphorus (mg/kg)	−0.1725	0.2992	3.21	0.0156 *	0.0453
Total potassium (g/kg)	0.6069	−0.1993	7.4	0.0001 **	0.2089
Available potassium (mg/kg)	−0.3577	−0.241	2.71	0.0318 *	0.0417

CV, cumulative variance. * Significant at *p* < 0.05; ** significant at *p* < 0.01.

## Supplementary Materials

The following are available online at https://www.mdpi.com/1660-4601/17/2/434/s1, Figure S1: Scanning pictures of new roots. Rocky trough (I), Rocky surface (II), Rocky gully (III), Rocky soil surface (IV), Rocky pit (V), Soil surface (VI); Lowercase Arabic numerals represent duplicates.
