# Supplementary material for: Influence of Heterogeneous Karst Microhabitats on the Root Foraging Ability of Chinese Windmill Palm (Trachycarpus fortunei) Seedlings
Source: Int J Environ Res Public Health. 2020 Jan 9;17(2):434. doi: 10.3390/ijerph17020434 (PMC7014210; doi:10.3390/ijerph17020434)
Supplement: Supplementary file 1 [file ijerph-17-00434-s001.pdf]

International Journal of
Environmental Research and Public Health

MDPI

Article

Influence of Heterogeneous Karst Microhabitats on the Root Foraging Ability of Chinese Windmill Palm (*Trachycarpus fortunei*) Seedlings

Yingying Liu, Xiaoli Wei *, Zijing Zhou, Changchang Shao and Shicheng Su

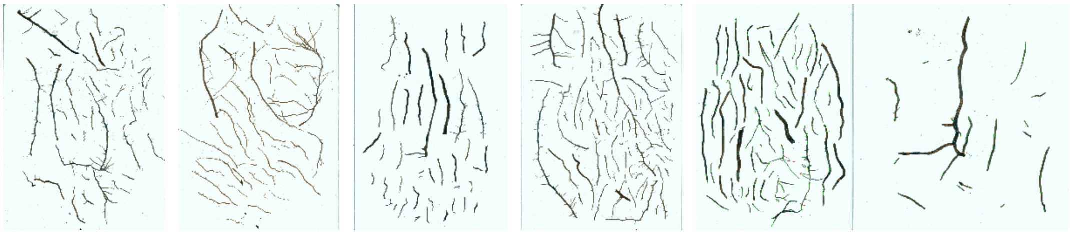

Ⅰ-1 Ⅰ-2 Ⅰ-3 Ⅰ-4 Ⅰ-5

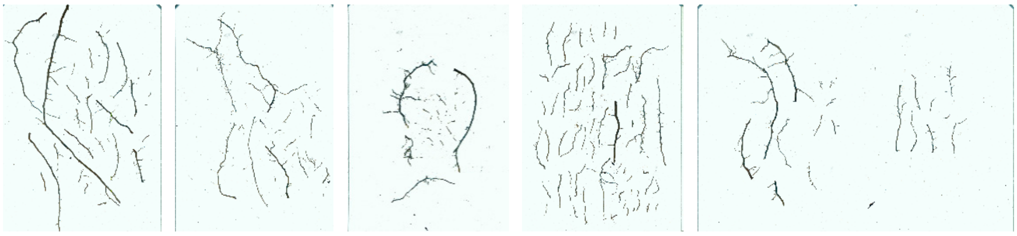

Ⅱ-1 Ⅱ-2 Ⅱ-3 Ⅱ-4 Ⅱ-5

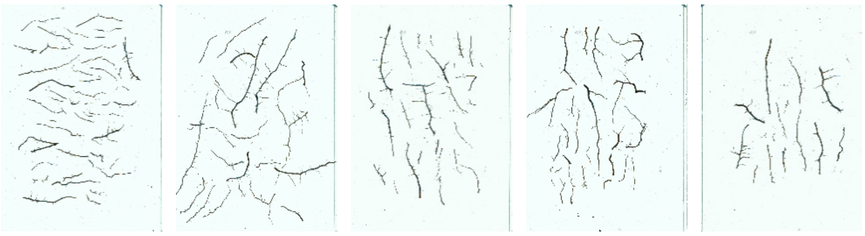

Ⅲ-1 Ⅲ-2 Ⅲ-3 Ⅲ-4 Ⅲ-5

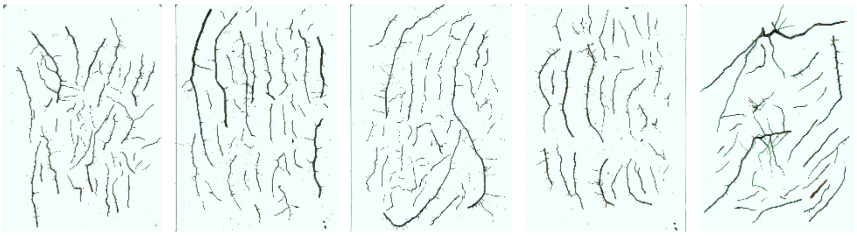

Ⅳ-1 Ⅳ-2 Ⅳ-3 Ⅳ-4 Ⅳ-5

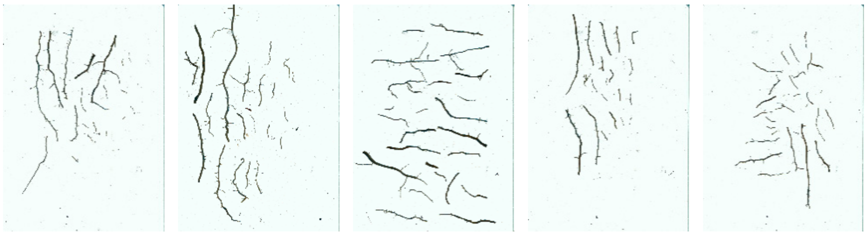

V-1 V-2 V-3 V-4 V-5

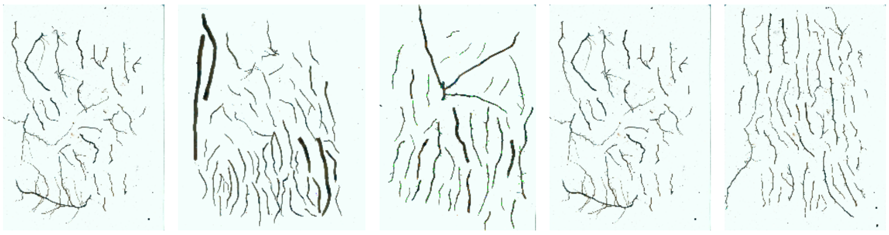

VI-1 VI-2 VI-3 VI-4 VI-5

Figure S1. Scanning pictures of new roots. Rocky trough (I), Rocky surface (II), Rocky gully (III), Rocky soil surface (IV), Rocky pit (V), Soil surface (VI); Lowercase Arabic numerals represent duplicates.

© 2020 by the authors. Submitted for possible open access publication under the terms and conditions of the Creative Commons Attribution (CC BY) license (http://creativecommons.org/licenses/by/4.0/).
